# Rethinking Trust in Synthetic Health Data: Lessons From 7 European Research Initiatives

**DOI:** 10.2196/83369

**Published:** 2026-04-29

**Authors:** Jens Declerck, Dipak Kalra, Antti Airola, Ahmed Youssef Ali Amer, Christos Chatzichristos, Maria del Mar Mañu Pereira, Bruno M. de Brito Robalo, Francesco Ghini, Alberto Gutierrez-Torre, Sem Hoogteijling, Susanne Hultsch, Jan Ramon, Sara Reidel, Francesco Regazzoni, Luís Silva, Inês Silveira, Tsekeridou Sofia, Christophe Maes

**Affiliations:** 1 The European Institute for Innovation through Health Data Oosterzele Belgium; 2 Unit of Medical Informatics and Statistics Department of Public Health and Primary Care The University of Ghent Ghent Belgium; 3 Department of Computing University of Turku Turku Finland; 4 Innovative Medicine Johnson & Johnson Beerse Belgium; 5 Department of Electrical Engineering KU Leuven Leuven Belgium; 6 University Hospital Vall d'Hebron Barcelona Spain; 7 Department of Obstetrics and Gynaecology Erasmus MC University Medical Center Rotterdam The Netherlands; 8 Laboratory of Translational Research Reggio Emilia Italy; 9 Barcelona Supercomputing Center Barcelona, Catalonia Spain; 10 Department of Neurology and Neurosurgery University Medical Center Utrecht Brain Center Utrecht The Netherlands; 11 VEIL.AI Helsinki Finland; 12 Institut national de recherche en sciences et technologies du numérique Lille France; 13 Vall d'Hebron Research Institute University Hospital Vall d'Hebron Barcelona Spain; 14 BarcelonaTech (UPC) Universitat Politècnica de Catalunya Barcelona Spain; 15 University of Amsterdam Amsterdam The Netherlands; 16 Università della Svizzera italiana Lugano, Ticino Switzerland; 17 Laboratory for Instrumentation, Biomedical Engineering and Radiation Physics NOVA School of Science and Technology Caparica Portugal; 18 Netcompany BELUX Athens Greece

**Keywords:** health data quality, synthetic data generation, health data, data quality dimensions, privacy, project

## Abstract

Synthetic data generation (SDG) structured health data is increasingly promoted as a solution to longstanding barriers in health data access. It is offering the promise of privacy-preserving data reuse for research, innovation, and policy. Despite rapid technical advances, the adoption of synthetic health data in real-world settings remains limited. Shaped by challenges around data quality, representativeness, infrastructure readiness, trust, and legal uncertainty, this viewpoint draws on experiences from 7 European research initiatives within the HealthData4EU cluster to reflect on how SDG is being operationalized in practice. It synthesizes cross-project insights to highlight recurring methodological and governance tensions and to examine their implications for trust and responsible use. The analysis argues that trustworthy SDG cannot be achieved through technical optimization alone but requires alignment between evaluation practices, upstream data stewardship, regulatory clarity, and sustained stakeholder engagement. Addressing these conditions is essential for moving synthetic data from experimental pilots toward a credible and sustainable component of European health research ecosystems.

## Introduction

As health care systems are largely digitized and become increasingly data-driven, access to large-scale, high-quality data is essential for clinical research [1-3], digital health innovation [4], and evidence-based policymaking [5]. However, accessing patient-level health data remains a persistent challenge [6]. Privacy regulations [7], data quality issues [8,9], and data fragmentation [10] continue to limit data access and sharing, especially in cross-organizational studies [9,11]. These barriers not only slow innovation but also risk underrepresenting certain diseases and populations, such as rare conditions, in secondary data use. As a result, many promising analytical and clinical applications struggle to move beyond pilots.

Synthetic data generation (SDG) has emerged as a promising response to the barriers limiting access and reuse of patient-level health data [12]. By producing synthetic datasets that resemble the statistical and structural properties of real health data, without exposing personal identities, SDG can drive innovation while maintaining privacy [7]. It enables safer collaboration across different types of health data (eg, electronic health records, genomics, medical imaging) and offers value in areas such as artificial intelligence (AI) development [7,13], rare disease research [14], and model testing [13,15]. The main aim of SDG is to retain the essential characteristics of original datasets while retaining privacy (in most use cases). These can be grouped into quantitative [16] (eg, statistical metrics, analytical patterns, signal-based features), qualitative [16] (eg, expert-driven assessments), and domain-specific attributes [17] that depend on the data type and specific use case. Conceptual work has also emphasized the importance of incorporating domain knowledge into SDG processes to improve realism and relevance for clinical applications [18]. Despite this growing interest, the adoption of synthetic health data in real-world research and clinical environments remains limited [12,19]. Concerns persist regarding data quality, representativeness, interpretability, and legal status [20,21], as well as the uncertainty about how synthetic data exceed what current methods and data infrastructures can deliver [13]. This gap between promise and practice has contributed to skepticism among multiple stakeholders, for whom trust in data sources is essential [22]. This lack of trust is partly explained by the focus of SDG literature on technical methods and evaluation metrics [7,22,23] while giving limited attention to the organizational, legal, and sociotechnical conditions that shape real-world adoption [7,22].

Several EU-funded initiatives are currently testing SDG in real-world health research settings, all facing a shared question: how can synthetic health data be made trustworthy, useful, and safe? Despite this activity, there has been limited cross-initiative reflection on how these challenges are being addressed in practice.

The aim of this viewpoint is to examine why trust in synthetic health data remains fragile despite growing technical maturity and to reflect on the nontechnical conditions required for its responsible adoption. This article is intended for health data researchers, clinicians, data stewards, infrastructure developers, regulators, and policymakers engaged in secondary use of health data across Europe. This article provides a viewpoint informed by experiences from 7 European research initiatives within the HealthData4Eu cluster that work with structured synthetic health data. It synthesizes cross-project insights to highlight recurring challenges related to data quality, interoperability, regulatory alignment, and stakeholder acceptance and reflects on their implications for trust, governance, and responsible use of synthetic data in European health research.

### Basis of This Viewpoint

#### Scope and Context of the Included Projects

This viewpoint is informed by 7 EU-funded projects that constitute the HealthData4EU cluster, a Horizon Europe initiative focused on advancing SDG and secure data use in health research across Europe. Collectively, these projects address complementary aspects of the synthetic data landscape, including data generation methods, federated and privacy-preserving architectures, secure data sharing, and AI-enabled clinical applications. All operate within a shared European policy and funding framework while engaging with diverse clinical and institutional contexts relevant to the European Health Data Space.

The included initiatives represent the full set of synthetic data projects participating in the HealthData4EU cluster at the time of writing. While they operate within a shared European policy and funding framework, they span a wide range of clinical domains, data modalities (eg, tabular, imaging, longitudinal, multimodal data), and technical approaches. This diversity within a coordinated structure provides a useful basis for reflecting on how SDG is being operationalized across health care settings and where common challenges and tensions emerge. An overview of the participating initiatives with their clinical domains is provided in Table 1.

**Table 1:** Overview of projects, clinical domains, and example use cases involving synthetic data.

Project	Clinical domain(s)	Example use cases
AISym4MED	Neurological and chronic diseases (ie, diffuse large B-cell lymphoma, lung cancer, multiple myeloma, Alzheimer disease, type 2 diabetes, breast cancer)	Synthetic data for multimodal applications Support for AI^a^ model testing
FLUTE	Oncology (prostate cancer)	Risk stratification Federated AI validation for prostate cancer diagnosis
PHASE IV AI	Oncology and neurology (ie, lung cancer, prostate cancer, ischemic stroke)	Early lung cancer detection Synthetic magnetic resonance imaging for stroke and model testing
PHEMS	Pediatric diseases (ie, congenital cardiac conditions, sepsis, hemophilia)	Sepsis prediction in pediatric intensive care units Hemophilia care optimization
SECURED	Multiple domains (i.e.ie, mammography, histopathology, chest radiography, cardiotocography)	Privacy-preserving SDG^b^ Federated AI tool development
SYNTHEMA	Rare hematological diseases (ie, sickle-cell disease, acute myeloid leukemia)	Synthetic datasets for rare disease research Federated validation
SYNTHIA	Multimodal and personalized medicine	Benchmarking and validation of SDG methods across diverse clinical scenarios

^a^AI: artificial intelligence.

^b^SDG: synthetic data generation.

The insights synthesized in this paper draw on cross-project engagement conducted during the active lifetime of the initiatives. These insights were informed by cluster-level workshops, project presentations, and recurring exchanges among project teams and by review of the project documentation during the active implementation phase. Importantly, these interactions took place while projects were still ongoing, allowing challenges and emerging practices to be discussed as they arose rather than retrospectively. The exchanges involved multidisciplinary contributors, including technical developers, clinicians, data stewards, infrastructure providers, and legal and ethical experts.

While these insights are not derived form a formal empirical study, they reflect sustained, longitudinal engagement with projects that are actively developing and validating the real-world use of synthetic health data within regulated clinical and research environments. To improve transparency and address requests for additional project-level context, we have incorporated a table in Multimedia Appendix 1 providing a descriptive overview of the included projects.

### Lessons From 7 SDG Projects

#### A Shared Landscape of Methodological Tensions

Across projects, SDG is not pursued as a single technical task but as a complex sociotechnical intervention embedded in heterogeneous health systems [22]. In practice, SDG sits at the intersection of machine learning, clinical data infrastructures, regulatory governance, and institutional trust.

Despite differences in disease focus, data modalities, and maturity, projects consistently encounter several recurring challenges that shape how SDG is operationalized in practice. Across the initiatives examined in this paper, 3 themes emerged repeatedly: the absence of shared consensus on how synthetic data quality should be defined and evaluated, the limitations of existing data infrastructures to support reliable SDG workflows, and uncertainty surrounding the regulatory and governance status of synthetic data. These challenges reflect the structural characteristics of health data ecosystems, where technical innovation must coexist with legal safeguards, clinical accountability, and fragmented infrastructures. Understanding SDG through these challenges helps explain why synthetic data adoption remains difficult to scale beyond pilot environments.

#### Data Quality Without Consensus

A central tension concerns how “data quality” in synthetic health data is defined and demonstrated. In practice, data quality is assessed through overlapping but fragmented lenses: statistical fidelity, analytical or clinical utility, and privacy protection. These dimensions are widely acknowledged across initiatives, yet there is no shared understanding of how they should be balanced, prioritized, or interpreted in different contexts [24].

Existing tools and metrics often assume that real-world source data are themselves unbiased, complete, and representative [3,6,8,9]. This assumption is rarely valid in health systems shaped by structural inequities, fragmented data capture, and variable clinical practices across institutions and regions. When real data reflect structural inequities or incomplete capture, synthetic data derived from them may inherit the same distortions [20].

As a result, SDG risks reinforcing existing biases rather than mitigating them. While synthetic data are frequently promoted as a mechanism for improving representativeness or enabling rare disease research, representational properties are frequently assessed primarily at the level of the resulting dataset [6]. In practice, however, representativeness is shaped not only by the quality of the synthetic generation process but also by the characteristics of the underlying source data and the transformations applied during the data lifecycle (eg, data extraction, harmonization, mapping to common data models, other extraction transfer load steps) [25]. These can introduce or amplify biases by selectively filtering variables, standardizing heterogeneous data sources, or excluding smaller subpopulations. These upstream influences may remain difficult to detect when evaluation focuses primarily on statistical similarity. As a result, population coverage and the potential impact of data preparation processes on representation may receive less attention in current evaluation practices.

In this sense, data quality cannot be reduced to statistical resemblance alone. We argue that there is a need for a more explicit data quality assessment at source and across the data lifecycle. In that sense, results need to be interpreted in relation to the populations, decisions, and governance contexts in which synthetic data are intended to be used.

#### Infrastructure as a Bottleneck to Trust

Federated and decentralized SDG architectures are frequently presented as solutions to privacy and governance constraints [13]. In practice, infrastructure maturity at data sources emerges as a critical limiting factor. Heterogeneous hospital IT systems, inconsistent data models, and uneven data governance practices complicate federated training and validation. These challenges affect not only technical integration but also the reliability of data inputs and the feasibility of data quality assessments.

Interoperability challenges persist even when projects adopt common data models such as the Observational Medical Outcomes Partnership Common Data Model. Differences in local coding practices, laboratory workflows, and semantic interpretation (eg, variation in LOINC [Logical Observation Identifiers Names and Codes] mappings for similar laboratory measurements) require substantial upfront coordination. These nuances complicate federated validation and cross-site comparability, reinforcing that standardization alone does not eliminate heterogeneity in real-world health data. Within the HealthData4EU cluster, several projects reported that aligning local coding practices and laboratory mappings across institutions required substantial preprocessing effort before federated SDG or validation could be performed.

These infrastructural constraints shape what forms of SDG are feasible and who can participate, reinforcing a gap between technical potential and institutional readiness.

### Trust, Transparency, and Regulatory Uncertainty

#### Trust is Not Guaranteed by Compliance

Most initiatives emphasize compliance with data protection and ethical frameworks. While compliance is necessary, it is not sufficient to generate trust. Trust emerges through transparency, interpretability, and meaningful stakeholder engagement across the SDG lifecycle [22].

In practice, these dimensions are unevenly operationalized. Some initiatives invest in dataset documentation, model cards, and participatory validation involving clinicians or data stewards. Others limit engagement to formal approval processes or internal review. This variability affects adoption, particularly in clinical and regulatory contexts where accountability and explainability are essential.

In the European context, this includes emerging obligations under the EU Artificial Intelligence Act, specific for high-risk medical AI systems, where the provenance and validation of training data, including synthetic data, may become subject to increased scrutiny.

#### The Unresolved Legal Status of Synthetic Data

Regulatory ambiguity remains one of the most significant barriers to uptake. Synthetic data are often assumed to fall outside data protection regulation, yet institutions frequently adopt conservative interpretations, particularly of rare diseases or small cohorts [26]. The absence of harmonized guidance leaves data controllers and ethics boards navigating uncertainty case by case.

Clarification is particularly needed in relation to General Data Protection Regulation (GDPR) anonymization thresholds, the interaction with the EU Artificial Intelligence Act, and national interpretations by data protection authorities and ethics committees.

This uncertainty raises a fundamental but often unspoken question: what is the value of synthetic data if it cannot be confidently reused beyond its original context? If synthetic datasets remain confined to local pilots due to legal or institutional caution, their potential to support cross-border research, reproducibility, and capacity building is limited. Addressing reusability therefore becomes central to the trust debate, linking legal clarity, documentation, and transparent validation of the broader promise of synthetic data as a shared research asset rather than a one-off technical item.

#### Emerging Responses and Their Limits

Encouragingly, initiatives are beginning to respond through federated validation pipelines, benchmarking libraries, interoperability standards, and public-facing platforms. However, these responses remain fragmented, and no shared consensus has yet emerged on what constitutes trustworthy SDG across contexts.

#### Reflections and Recommendations

The experiences discussed in this article suggest that SDG in health research has reached a point where technical feasibility is no longer the primary bottleneck. Instead, progress increasingly depends on how synthetic data are evaluated, governed, communicated, and trusted in practice.

First, there is a need to reframe how success in SDG is defined and assessed. Much of the current emphasis remains on demonstrating technical performance, such as statistical similarity or model accuracy. While these measures are important, they do not on their own indicate whether synthetic data are appropriate for research or operational contexts. In practice, synthetic datasets may serve different purposes (eg, exploratory analysis, educational use, or hypothesis testing). These uses imply different expectations regarding the level of fidelity, reliability, and validation required. Clarifying the intended context of use can therefore help guide how synthetic datasets are evaluated and interpreted. This does not imply that all potential analytical applications must be evaluated in advance, but rather that evaluation strategies should be transparent about the contexts for which synthetic datasets are considered suitable. At the same time, general evaluation metrics remain essential to characterize the overall fidelity and privacy properties of synthetic datasets. In many cases, findings derived from exploratory analysis using synthetic data may subsequently require validation using the original data sources where appropriate access mechanisms exist.

Second, greater attention should be given to data stewardship and governance upstream of SDG. Synthetic data reflects the characteristics and limitations of the infrastructures from which it is generated. Without robust practices for data quality management, interoperability, and documentation at source, SDG risks perpetuating existing shortcomings rather than addressing them. Aligning SDG initiatives with broader efforts to strengthen health data infrastructures would therefore increase both the reliability of synthetic outputs and institutional confidence in their reuse.

Third, addressing the legal uncertainty surrounding synthetic health data is essential for wider adoption. In the absence of shared guidance, institutions often adopt cautious positions that limit reuse, particularly in sensitive or cross-border contexts. Developing common interpretations, best practices, or certification pathways that clarify when synthetic data can be considered sufficiently anonymized would provide a more stable foundation for decision-making. Such approaches would not eliminate the need for contextual oversight, but they would reduce fragmentation and support more consistent application of safeguards.

Finally, trust building must be recognized as an active and ongoing process. Trust does not arise automatically from compliance with legal or ethical requirements nor from technical sophistication alone. It depends on transparency, interpretability, and the ability of stakeholders to understand how synthetic data were generated, evaluated, and intended to be used. Embedding mechanisms such as clear documentation, accessible validation summaries, and engagement with clinicians, data stewards, and other end users can support accountability and enable more nuanced judgments about risk and value.

Overall, these reflections point to a broader shift in focus: from demonstrating that synthetic data can be generated to establishing the conditions under which it can be used responsibly, communicated transparently, and governed consistently. Advancing SDG along this path will require coordination across technical, institutional, and regulatory domains. If achieved, synthetic data can become not only a powerful analytical tool but also a credible and trusted component of health research ecosystems.

## Appendix

Multimedia Appendix 1 Overview of all HealthData4EU projects, their objectives, data modalities, use cases, and methodological challenges.

## Abbreviations

AI: artificial intelligence
GDPR: General Data Protection Regulation
LOINC: Logical Observation Identifiers Names and Codes
SDG: synthetic data generation
