# Supplementary material for: Rethinking Trust in Synthetic Health Data: Lessons From 7 European Research Initiatives
Source: J Med Internet Res. 2026 Apr 29;28:e83369. doi: 10.2196/83369 (PMC13173070; doi:10.2196/83369)
Supplement: Multimedia Appendix 1 [file jmir_v28i1e83369_app1.docx]

| 1. **Methodological challenges addressed**   *Understand what problems the project is trying to solve and how it technically approaches synthetic data generation.* | | | | | | | | |
| --- | --- | --- | --- | --- | --- | --- | --- | --- |
| **Item** | **SYNTHEMA** | **PHASE IV AI** | **SECURED** | **FLUTE** | **PHEMS** | **AI SYM4Med** | **SYNTHIA** |  |
| Project Name | Synthetic generation of haematological data over federated computing frameworks | Privacy compliant health data as a service for AI development | Scaling up secure processing, anonymization and generation of health data for EU cross border collaborative research and innovation | Federate learning and multi-party computation techniques for prostate cancer | Pediatric Hospitals as European drivers for multi-party computation and synthetic data generation capabilities across clinical specialities and data types | AI-integrated medical device platform for federated and synthetic data in healthcare | Synthetic Data Generation Framework for Integrated Validation of Use  Cases And Ai Healthcare Applications |  |
| Funding | Horizon Europe | Horizon Europe | Horizon Europe | Horizon Europe | Horizon Europe & UK RI | Horizon Europe | Innovative Health Initiative |  |
| Duration | 1 December 2022 – 30 November 2026 | 1 October 2023 – 30 September 2026 | 1 January 2023 – 31 December 2025 | 1 May 2023 – 31 March 2026 | 1 May 2023 – 30 April 2026 | 1 October 2023 – 30 September 2026 | 1 September 2024 – 31 August 2029 |  |
| Main Objective(s) | - Establish a cross-border health data hub for rare haematological diseases (RHDs). - Develop and validate AI-based techniques for clinical data anonymisation and synthetic data generation (SDG). - Address data scarcity and fragmentation. - Support GDPR-compliant research through synthetic data. | - Develop improved, privacy-preserving data synthesis methods for multiple data formats (e.g., EHRs, medical images). - Provide easy-to-use, configurable data services for AI developers to access decentralized, de-identified data via multi-party computing. - Enable anonymous data provision on demand or from temporary repositories. - Establish a data marketplace for sharing and monetizing data, including incentive-based contributions. | - Enable data providers, researchers, and innovators do develop AI-based analytics. - Foster health data innovation across Europe. - Provide a secure, trusted innohub environment using Secure Multiparty Computation (SMPC) and Homomorphic Encryption (HE). - Offer tools and services for anonymization, de-anonymization, SPMC, and debiasing health data. | - Advance privacy-preserving, cross-border healthcare data use for AI-driven healthcare. - Innovate in secure computing with novel methods for secure multi-party computation and federated learning. - Build a secure platform for developing, testing, and deploying federated AI solutions using real and synthetic health data. - Contribute to HL7 FHIR standards and GDPR-compliant federated learning guidelines. - Integrate the FLUTE platform with health data hubs in three countries to clinically validate federated AI tools for diagnosing prostate cancer. | - Build an open ecosystem for secure, cross-border collaboration using anonymized and synthetic health data. - Demonstrate the impact of federated and synthetic data via paediatric studies (cardiac care, sepsis, haemophilia). - Engage stakeholders (hospitals, researchers, SMEs, public) via hackathons, webinars, and online tools. - Develop a sustainable commercial plan to support long-term health data services. - Pioneer a decentralized ecosystem for paediatric synthetic health data in full regulatory compliance. | - Provide a platform for data engineers, clinicians, and researchers to access a trustworthy, controlled synthetic dataset system. - Combine anonymization techniques with privacy-aware mechanisms and trust tracking. - Ensure data quality through fairness, ethical validation, and context-aware data controls. - Use federated technology to synthesize unidentifiable data across closed borders while respecting GDPR and security regulations. | - Support responsible use of synthetic data in healthcare. - Advance SDG techniques across different modalities (e.g., genomics, imaging) to produce realistic, multimodal, and longitudinal synthetic data. - Establish transparent benchmarking tools for comparing SDG methods. - Define robust evaluation metrics and contribute to the standardization of assessment frameworks for synthetic data quality and utility. |  |
| Target Healthcare Domain(s) | Rare haematological diseases  *Sickle-cell disease and acute myeloid leukaemia* | Oncology and neurology:  *Lung cancer, prostate cancer, and ischaemic stroke* | Multiple data domains:  *Mammography, histopathological imaging, chest X-Ray, cardiotocography* | Paediatric diseases:  *Congenital cardiac conditions, sepsis in paediatric intensive care units (PICUs), and haemophilia.* | Neurological and chronic diseases:  *Diffuse large B-cell lymphoma, lung cancer, multiple myeloma, Alzheimer’s disease, type II diabetes, and breast cancer.* | Multiple disease domains:  *Ophthalmology (diabetic retinopathy), neurology (epilepsy and, advanced Parkinson’s disease), cardiology (congestive heart failure), and respiratory medicine (chronic respiratory disease monitoring)* | Multimodal healthcare applications with a focus on personalized medicine and the practical utility of synthetic data across diverse clinical contexts. Data modalities include Lab results, clinical notes, genomics, imaging, m-health data |  |
| Use Cases | - Sickle-cell disease (SCD) - Acute myeloid leukaemia (AML) | - Early lung cancer detection (EHR & CT) - Prostate cancer risk stratification (EHR) - Synthetic MRI for ischaemic stroke | - Medical personnel training - Support in brain surgery - Support in training models for sensorized beds in pediatric hospitals - Research on genomic data   Supplementary data for analysis | - Cardiology patient benchmarking - Sepsis prediction in PICUs - Haemophilia care optimization. | - Diabetic retinopathy - Advanced Parkinson’s disease - Epilepsy - Congestive heart failure (CHF) - Chronic Obstructive Pulmonary Disease (COPD) | - Diabetic retinopathy detection and prediction - Retrospective extraction of clinical knowledge - Advanced Parkinson disease prediction - Epilepsy surgery planning - Chronic heart and respiratory disease monitoring | - Benchmarking and validation of synthetic data across multimodal personalized medicine scenarios |  |
| Generation Techniques | - Variational autoencoders (VAE) for genomic data - Segmentation-based generative models for cytological imaging - Synthetic feature-level generation for radiomics | - Marginal-based methods (for tabular data) - Generative Adversarial Networks (GANs) - Variational Autoencoders (VAEs) - Diffusion models | - Generative Adversarial Networks (GANs) - Variational Autoencoders (VAEs) - Diffusion models | - UMAP-based instance generation, involves iterative feature removal and imputation - GANs, VAEs, and diffusion models to generate synthetic data from random latent space | - Synthetic data generation from EHR and medical images using VEIL.AI anonymization engine | - GANs for tabular and time-series data - Diffusion models for image generation | - Not identified yet |  |
| Synthetic Data Type | - Tabular clinical data - Medical imaging (MRI, Whole Slide Imaging) - Genomic data (e.g., NGS, RNA-seg) - Metabolomic profiles | - Tabular data - Medical images - Longitudinal data | - Tabular data - Time-series data - Medical images | - Tabular data - Image data - Multimodal data (combined sources) | - Tabular data - Time-series data - Medical images - Multimodal data (combined sources) | - Tabular data - Time-series data - Medical images - Multimodal data (combined sources) | - Tabular data - Imaging data - Sensory time-series data - Genetic data |  |
| Success Indicators (e.g. evidence of project uptake or stakeholder trust) | - Completion of validated synthetic datasets for SCD and AML - Integration of tools and datasets into a federated infrastructure - Interest and collaboration from other projects (e.g., HealthData4EU cluster) - Peer-reviewed publications, training programs, and GDPR compliance alignment | - Development of data quality and privacy evaluation metrics - Validation of SDG methods through replication studies - Demonstrated success in use cases using synthetic data - Health Data Hub services development and third-party uptake | - Performance of predictive models evaluated - Clinical relevance demonstrated through use-case alignment - Data specific evaluation metrics - Feedback from doctors (e.g., radiologists) in the project | - Improved clinical prediction of aggressive prostate cancer - Reduction in unnecessary biopsies and healthcare cost savings - Multi-national validation of federated AI toolset - Platform adoption across three national health data hubs - Proven synthetic data generation and usage in real-world development | - Deployment of federated nodes across partner hospitals - Enhanced ML model accuracy in paediatric cardiac care, sepsis, and haemophilia - High-quality synthetic data with demonstrated utility - Strong stakeholder engagement hospitals (hospitals, SMEs, public) through events - Public dissemination of methods and tools via open-source platforms | - Performance of predictive models evaluated - Clinical relevance demonstrated through use-case alignment | - SYNTHIA platform development - SDG models and datasets for at least two diseases developed and evaluated - Benchmarking workflows and guidance for synthetic data regulation published - Models fine-tuned for federated settings and integrated into the SYNTHIA catalogue |  |
| Challenges Faced | - Data fragmentation across sites and countries - Lack of publicly available datasets - Ethical limitations on sharing real patient data | - Unclear regulatory boundaries on what qualifies as “anonymous” synthetic data - Restrictions on cross-border data sharing - Diverse data quality metrics with no standardized consensus - Compatibility challenges of SDG methods with federated infrastructures | - Lack of publicly available datasets and lack of tagged data when available - Ethical limitations on sharing real patient data - Difficulty on the evaluation of synthetic data with metrics | - Addressing strict privacy and security expectations for sensitive health data - Computational demands of advanced SMPC and federated AI models - Difficulty ensuring representativeness and utility of synthetic data, particularly for rare cases | - Regulatory ambiguity; unclear distinctions between synthetic and anonymized data in hospital policies - Complex integration of federated learning into diverse hospital IT systems - Data heterogeneity; inconsistent quality, structure, and completeness across partner institutions | - Data harmonization across clinical sites | - Variation in data models and formats across institutions - Interoperability issues in deploying SDG models over federated networks |  |
| 1. **Approaches to data quality**   *Focuses on how the project defines, ensures, and validates the trustworthiness of synthetic data* | | | | | | | | |
| **Item** | **SYNTHEMA** | **PhaseIV AI** | **Secured** | **Flute** | **Phems** | **AI Sym4Med** | **Synthia** |  |
| Data Quality Dimensions Emphasised | - Statistical fidelity (alignment with real-world distributions) - Clinical utility (relevance for medical decision-making) - Privacy protection (anonymisation, GDPR alignment) - Data completeness, consistency, accuracy, and integrity | - Statistical fidelity (alignment with real-world distributions) - Clinical utility (relevance for medical decision-making) - Privacy protection (anonymisation, GDPR alignment) | - Statistical fidelity (alignment with real-world distributions) - Clinical utility (relevance for medical decision-making) - Privacy protection (anonymisation, GDPR alignment) | - Privacy protection - Standardi-zation / nor- malisation of data from different data owners | - Statistical fidelity - Clinical/Use Case utility - Privacy | - Fidelity - Diversity - Utility - Privacy |  |  |
| Quality Definition – Operationalisation | - Alignment with OMOP CDM - Validation using real-world data benchmarks - Clinician-supervised thresholds and review processes | - Metrics comparing synthetic to real data along key axes (fidelity, utility, and privacy) - Replication studies (same analysis applied to both real and synthetic datasets, results compared for consistency) - Use of differential privacy techniques to guarantee controlled disclosure risk | - Use of differential privacy techniques to guarantee controlled disclosure risk - Compare synthetic to real data based on distributional features and data specific metrics | - Compare synthetic to real data based on distributional features - Units / domains the same across data owners - Statistical privacy (e.g., differential privacy) | - Defined resemblance levels   *URA (Univariate resemblance analysis)*  *MRA (Multivariate resemblance analysis)*  *DRA (Dimensional resemblance analysis)*  *DLA (Data labelling analysis)*   - Utility measured by   *TRTR (Train on Real, Test on Real)*  *TSTR (Train on Synthetic, Test on Real)*   - Privacy risks measured via   *SEA (Similarity Evaluation Analysis)*  *MIA (Membership Inference Attack)*  *AIA (Attribute Inference Attack)* | - Same metrics used for real and synthetic data - Higher SQ quality means greater similarity to real data - Focus on statistical, struct al, and clinical alignment | - Quality checks applied to both real (RD) and synthetic data (SD) - RD evaluated for clinical relevance and model compatibility - SD assessed for fidelity to RD characteristics and clinical logic |  |
| Validation Strategies | - Uses SAFE framework   *Model training and evaluation*  *Federated validation*  *FL platform integration* | - Uses standard evaluation metrics - Validated by domain experts | - Uses standard evaluation metrics - Validated by domain experts - Prediction accuracy | - Prediction accuracy - Synthetic data distributional features - Expert validation | - Applies previously defined evaluation metrics - Aligned with project-specific quality criteria | - Standard evaluation metrics applied - Supported by expert validation | - Evaluation by domain experts - Includes technical, statistical, and analytical methods |  |
| Custom Tools – Metrics | - SAFE federated validation pipeline - Custom clustering (e.g., Dirichlet) - Privacy risk scoring tools | - Tune insight federated infrastructure - Built-in quality metrics - Tools for generating EHR and medical images | - Open-source Python libraries (e.g., SynthVal) | - Libraries for DNN - Libraries for privacy accounting | - Uses open-source tools (e.g. SDMetrics, syntheval) | - Open-source Python libraries (e.g., PyMDMA, seege_) | - Tools not specified yet |  |
| 1. **Strategies for trust and acceptance**   *Captures how the project builds stakeholder trust, and measures acceptance* | | | | | | | | |
| **Item** | **SYNTHEMA** | **PhaseIV AI** | **Secured** | **Flute** | **Phems** | **AI Sym4Med** | **Synthia** |  |
| Stakeholder Engagement | - Clinical partners in over 10 Eu countries - Engagement with SCD and AML expert clinicians - Planned webinars for patients and healthcare professionals | - Inclusion of clinical and AI experts - Patient representatives involved - Stakeholder input gathered across multiple European countries | - Healthcare institutions and medical research centres contribute data - These partners also shape clinical research questions | - Surveyed patients to understand their interests as stakeholder - Involved data scientists in requirements analysis | - Active clinician involvement throughout the project - Close collaboration with hospital IT, legal, and data stewards - Participation in EU synthetic data initiatives - Engagement via hackathons and webinars for user feedback | - Community of Practice including clinicians, patients, and data experts | - Healthcare institutions and medical research centres contribute data - These partners also shape clinical research questions |  |
| Transparency Measures | - Shared cohorts across partners - Clear validation logic defined and documented | - Synthetic data includes metadata on data origin and quality | - Modeling methodologies available in the public deliverables - Open data based models available through SECURED’s Innohub - Code is open source | - To preserve privacy, synthetic data avoids including any meta-data referring to its origin / patients whose features it includes. - Code is open source - Methodology is documented | - Datasets include standardised metrics (e.g., resemblance, utility, privacy) - Basic generation metadata (e.g., model type, input variables) is provided - Full training configs are withheld to protect data security | - Each synthetic dataset is delivered with a model card and dataset card | - Plans to publish key deliverables - SYNTHIA platform intended for external public access |  |
| Ethical/Regulatory Alignment | - GDPR-compliant by design - Ethical approval required per clinical site - Local pseudonymisation and consent-based inclusion | - GDPR compliance prioritized - Aligned with AI act and upcoming EHDS regulation | - Compliant with GDPR and AI act - Adheres to local partner regulations | - Project is regulatory compliant | - Compliant with GDPR and AI act - Adheres to local partner regulations | - Follows GDPR and AI Act | - Compliant with GDPR and AI Act |  |
| 1. **Innovation and ecosystem contribution**   *Highlights broader contributions like tools, standards, or collaboration models shared with others* | | | | | | | | |
| Technical Innovations | - Cross-border ETL pipelines - Segmentation-based synthetic image generation - Federated validation architecture with privacy safeguards | - Synthetic data generation and evaluation pipelines - Development of the Health Data Hub | - Synthetic data generation methods and models - Privacy preserving tools and services - Development of the Secure and Trusted decentralized data processing Hub | - Scaling up of MPC and FL technology - Improvement of security of federated AI (next to privacy) | - Federated node infrastructure - SDG and evaluation pipelines - OMOP-compatible federated AI models - Privacy-preserving tools for paediatric rare disease research | - Python library for data quality evaluation (input and generated data) | - Novel model development and enhancement - Adaptation of models for federated networks (FN) - SYNTHIA platform and infrastructure development - Synthetic data generation and benchmarking pipeline |  |
| Interoperability Features | - OMOP Common Data Model (CDM) - SNOMED-CT, ORPHA, HPO, LOINC, ICD-10/11 - EU-RD platform common data elements | - OMOP Common Data Model (CDM) - Python machine learning libraries | - Python machine learning library integrations | - HL7 FHIR & DICOM standards - Conversion from OMOP - Python machine learning library integrations | - OMOP Common Data Model (CDM) - Python-based ML and federated learning libraries | - OMOP Common Data Model (CDM) - Python-based ML and deep learning libraries | - OMOP Common Data Model (CDM) - FLOWER federated network - Python libraries for ML, DL, and LLMs |  |
| Contribution to Ecosystem | - Harmonised rare haematological disease (RHD) datasets - Validated electronic Case Report Forms (eCRFs) - Tools and protocols for synthetic data validation | - Pipeline for synthetic data generation (SDG) and quality evaluation | - Anonymisation and Reidentification tools - Methodologies to asssess privacy leak through attacks to models - Generative models - SynthVal library for synthetic data quality assessment - Secure Multiparty Computation, Homomorphic Encription and Federated Learning frameworks - InnoHub platform - Generated synthetic data and models | - Use case in diagnosis of prostate cancer | - Paediatric rare disease cohorts - Federated training pipelines - Synthetic data resources | - Anonymisation toolkit - Generative models - pyMDMA library for data quality assessment | - SYNTHIA platform for SDG - Generated synthetic datasets and comparative studies - Full evaluation pipeline |  |
| Open Questions/Gaps | - Long-term scalability to other rare haematological diseases (RHDs) - Unclear exploitation model (research vs. commercial use) - Regulatory acceptance of synthetic data standards | - Regulatory acceptance of synthetic data and federated learning standards | - Scalability to broader use cases beyond project timeframe - Quantification of the privacy leak for all the developed models - Further improvement of model quality to better match real data - Metadata for traceability and trustability on the synthetic data | Too early to assess open challenges | - Scalability to broader use cases beyond project timeframe | - Need to quantify and certify privacy leakage - Challenges with semantic and ontology alignment (e.g., SNOMED CT, HPO, LOINC) - Difficulty maintaining multimodal coherence across data types | Too early to assess open challenges |  |
